# Evaluation of the Results of Group B Streptococcus Screening by MALDI-TOF MS among Pregnant Women in a Hungarian Hospital

**DOI:** 10.3390/pathogens9010001

**Published:** 2019-12-18

**Authors:** Marianna Ábrók, Petra Tigyi, Markus Kostrzewa, Katalin Burián, Judit Deák

**Affiliations:** 1 Institute of Clinical Microbiology, Albert Szent-Györgyi Clinical Center, University of Szeged, 6725 Szeged, Hungary; szalamandra@gmail.com (P.T.); burian.katalin@med.u-szeged.hu (K.B.); judithdeak@gmail.com (J.D.); 2 Bioanalytical Development, Bruker Daltonik GmbH, 28359 Bremen, Germany; Markus.Kostrzewa@bruker.com

**Keywords:** *Streptococcus agalactiae*, antibiotic susceptibility, ST-1, ST-17, MALDI-TOF MS

## Abstract

Pregnant women colonized by *Streptococcus agalactiae,* or group B streptococcus (GBS), are at an increased risk of premature delivery and stillbirth, and their neonates can be endangered by the development of an invasive GBS disease. In this study, the results of the GBS screening among pregnant women performed between 2012 and 2018 (n = 19267) are presented. For the GBS positive samples, the antibiotic susceptibility of the isolated strains was also tested (n = 3554). During the examined period, the colonization rate varied between 17.4% and 19.8%. The overall rate of erythromycin and clindamycin resistance in the GBS positive samples was 34.9% and 34.6%, respectively. The frequency of the erythromycin and clindamycin resistant strains showed an increasing tendency. An analysis of the MALDI-TOF MS spectra of 260 GBS isolates revealed that 46.5% of them belonged to either the ST-1 or the ST-17 sequence types, indicating a high prevalence of these potentially invasive GBS strains in our region. More than half of the strains identified as ST-1 (52.1%) proved to be resistant to erythromycin and clindamycin.

## 1. Introduction

*Streptococcus agalactiae* (*S. agalactiae*), also known as group B streptococcus (GBS), may cause serious infections in newborns, pregnant and postpartum women, and immunocompromised adults [1,2]. This Gram-positive β-haemolytic bacterium can cause urinary tract infections, fever, chorioamnionitis, endometritis, and puerperal sepsis in women [3,4,5], and it is among the leading causes of neonatal invasive diseases in the United States and Europe [5,6,7,8]. Approximately 10%–30% of pregnant women are colonized by GBS without any symptoms [6,9,10]. However, these women are at an increased risk of premature delivery and stillbirth, and their newborns can be exposed to the development of an invasive GBS disease [2,5]. Therefore, vaginal or rectal GBS colonization indicates intrapartum antibiotic prophylaxis. Matrix-assisted laser desorption/ionization time-of-flight mass spectrometry (MALDI-TOF MS) is an effective method for the identification of GBS strains at the species level [11,12]. Moreover, MALDI-TOF MS also proven to be applicable to detect the *S. agalactiae* sequence types ST-17 and ST-1 based on the characteristic peak-shifts present in their protein MS spectra [13]. Within *S. agalactiae*, various sequence types could be discerned by the multi locus sequence typing (MLST) method [11]. Among these MLST types, ST-17 is considered as highly virulent, and was found to be one of the most frequent agents of neonatal meningitis and GBS late-onset-disease (LOD) [13,14,15]. Furthermore, ST-1 has become another significant cause of invasive neonatal infections, since its emergence in the 1990s [13,15,16].

The aim of the present study was to analyze the results of the GBS screening among pregnant women and antibiotic susceptibility testing at the Albert Szent-Györgyi Clinical Center (University of Szeged, Hungary) between 2012 and 2018. We also report our experiences in the application of the rapid MALDI-TOF MS-based detection of ST-17 and ST-1 GBS.

## 2. Results

### 2.1. Colonization

During the examined period, vaginal or cervical samples of 19,267 pregnant women were screened for *S. agalactiae*, among which 3554 samples proved to be GBS positive (Table 1). The colonization rate showed only slight changes in the various years, and its value fluctuated between 17.4% and 19.8% (the mean value is 18.4%). Although much less samples (3566) were tested in the age group of 26–30 years than those (6510) in the age group of 31–35 years, the colonization rate was the same in both groups (19%), which was the highest rate compared with the other age groups (Table 1). At the same time, the colonization rate was higher than 10% in each age group.

### 2.2. Antimicrobial Susceptibility Testing

In the case of the 3554 GBS positive samples, the susceptibility of the isolated strains to penicillin, cefuroxime, vancomycin, erythromycin, clindamycin, and trimethoprim–sulfamethoxazole was also tested. All of the tested strains proved to be susceptible to beta-lactams (i.e., penicillin and cefuroxime), vancomycin, and trimethoprim–sulfamethoxazole. The overall frequency of the erythromycin and clindamycin resistant isolates were 34.9% and 34.6%, respectively, while co-resistance to the two antibiotics was detected in 33.2% of the GBS positive samples (Table 2). However, between the years 2012 and 2018, the frequency of the erythromycin and clindamycin resistant GBS strains increased from 29.2% and 30.2% to 39.7% and 38.7%, respectively, while the co-resistance level increased from 28% to 35.7% (Table 2). Only a low percentage of the isolated strains proved to be resistant to either erythromycin or clindamycin alone (i.e., 1.9% and 1.4%, respectively) (Table 2). Inducible clindamycin resistance was detected only in 6.5% of the isolated GBS strains.

### 2.3. ST-1 and ST-17 Detection

The presence of the protein peaks characteristic of the highly virulent invasive ST-1 and ST-17 clones was examined in the MS spectra of 260 randomly selected GBS strains isolated in 2017 and 2018. According to this analysis, 27.3% (71 strains) of the tested strains belonged to the ST-1 type, and 19.2% (50 strains) proved to be ST-17 (Table 3). While the proportion of the erythromycin and clindamycin co-resistant ST-17 strains (32%) did not differ significantly from that of the non-ST-1 and non-ST-17 strains (30.9%), 52.1% of the 71 ST-1 strains proved to be resistant to both antibiotics (Table 3). Moreover, all of these ST-1 strains were co-resistant to erythromycin and clindamycin. At the same time, 2% and 2.9% of the ST-17 and the non-ST-1 and non-ST-17 strains, respectively, proved to be resistant to clindamycin alone. The proportion of strains with induced clindamycin resistance was 4% (two strains) for the ST-17, 5.6% (four strains) for the ST-1, and 7.9% (eleven strains) for the non-ST-1 and non-ST-17 strains. Erythromycin resistance alone was not detected among the 260 tested strains.

## 3. Discussion

In our study, the GBS colonization level was found to be around 20% during the examined period. This value falls into the range of the colonization rates (i.e., from 6.5% to 36%) reported earlier for European countries [15,17], but it is somewhat higher than that reported recently in the neighboring country, Serbia (15%) [18]. At the same time, this relatively high colonization rate emphasizes the importance of the accurate GBS screening of pregnant women.

All of the GBS strains were susceptible to beta-lactams and vancomycin, which can be considered as a common pattern observed in several previous studies [19,20,21,22]. At the same time, a high level of resistance to erythromycin and clindamycin, and a clearly increasing tendency in the frequency of resistant strains has been observed over the years examined. Other studies have also reported the spreading of macrolide and lincosamide resistance among the GBS strains [23,24,25], and highlighted the need of susceptibility testing of colonizing GBS strains for an effective intrapartum antibiotic prophylaxis [10,11,12,13,14,15,16,17,18,19,20,21,22,23,24,25,26]. The majority of erythromycin and clindamycin resistant strains proved to be co-resistant to these antibiotics, and the majority of the detected clindamycin resistances were constitutive.

The method of Lartigue et al. [13] was used for the MALDI-TOF MS-based detection of the ST-17 and ST-1 strains. These GBS sequence types have frequently been associated with neonatal meningitis and invasive neonatal infections [15]. In our study, almost half (46.5%) of the examined *S. agalactiae* strains proved to be either ST-1 (27.3%) or ST-17 (19.2%) clones, indicating a high prevalence of these potentially virulent and invasive GBS strains in our region. It should be mentioned that, according to Lartigue et al. [13], the sensitivity and specificity of this identification method were 100% and 95%, respectively, for ST-1, and 100% and 98%, respectively, for ST-17.

ST-1 is thought to be the predominant colonizer in pregnant women, and found to be one of the most frequent clones in vaginal samples [15,27,28]. Our study also indicates a significant distribution of the ST-17 clone. Recently, Gajic et al. [18] found this sequence type as the most common clone in Serbian samples. Moreover, the rate of erythromycin and clindamycin co-resistance was found to be extremely high among the ST-1 clones (52.1%). Previously, Bergseng et al. [16] also found a strong association of ST-1 clones and erythromycin resistance.

To our knowledge, this study is the first analysis of the GBS screening results and the antibiotic susceptibility pattern of GBS strains colonizing pregnant women in our region, and the first data about the frequency of highly virulent ST-1 and ST-17 GBS clones in Hungary.

Our work demonstrates the value and usefulness of MALDI-TOF MS analysis in GBS screening, and reinforces that the examination of the MALDI-TOF MS spectra is a useful method to detect the potentially invasive ST-1 and ST-17 GBS clones. The high frequency of these clones in our samples indicates the importance of the accurate *S. agalactiae* screening of pregnant women, because almost half of GBS-positive pregnant women can be colonized by these highly virulent clones in our region.

## 4. Materials and Methods

### 4.1. Research Design and Samples

The study was conducted in the Albert Szent-Györgyi Clinical Center (University of Szeged, Hungary), and was approved by the institutional review board (no. 197/2017-SZTE). GBS screenings were performed on vaginal or cervical samples of all of the pregnant women aged from 14 to 58 years (n = 19,267) who attended at the clinical center to detect their GBS colonization status from January 2012 to July 2018. For each GBS positive sample, antibiotic susceptibility of the isolated GBS strain was tested (n = 3554). From the strains isolated from January 2017 to July 2018, 260 isolates were randomly selected and subjected to ST-1 and ST-17 detection.

### 4.2. GBS Screening of Pregnant Women

GBS screening was performed according to the CDC 2010 guideline [10] in each case. Samples were inoculated onto Columbia agar plates containing 5% sheep blood (bioMérieux, France), and incubated at 35–37 °C in 5% CO_2_. In parallel, a selective enrichment was also performed in modified Todd Hewitt broth (OXOID, England) supplemented with nalidixic acid (0.015 g/L) and colistin (0.010 g/L) at 35–37 °C in 5% CO_2_. After incubation for 18–24 h, the enriched broth was plated onto CHROMagar StrepB (CHROMagar, France) and subcultured at 37 °C under aerobic conditions for 18, 24, or 48 h. After incubation, the Columbia agar or CHROMagar StrepB plates were inspected to detect suspicious colonies. The presumptive GBS colonies were identified by MALDI-TOF MS or by traditional methods (i.e., colony morphology, beta-haemolysis, bacitracin resistance, CAMP test, and Lancefield-group B detection by latex agglutination test with Pastorex Strep Kit [Bio-Rad, France]).

### 4.3. MALDI-TOF MS Analysis

For the species level identification of the isolated bacterial colonies, a MALDI-TOF MS analysis was performed using a MALDI Biotyper system (Bruker Daltonik GmbH, Germany) in the positive linear mode across the *m/z* range of 2 to 20 kDa. For each spectrum, 240 laser shots at 60 Hz in groups of 40 shots per sampling area were collected. The spectra were analyzed by the MALDI Biotyper RTC 3.1 software (Bruker Daltonics, Germany), using the MALDI Biotyper Library 3.1. The ST-1 and ST-17 sequence types were identified by examining the MS spectra of the selected strains and detecting the presence of the 6250-Da protein specific to ST-1 or the 7625-Da protein specific to ST-17, according to the method described by Lartigue et al. [13].

### 4.4. Antibiotic Susceptibility Testing

Antimicrobial susceptibility was tested to penicillin, cefuroxime, vancomycin, trimethoprim–sulfamethoxazole, erythromycin, and clindamycin. The testing was performed by the disk diffusion method, according to the recommendations of The European Committee on Antimicrobial Susceptibility Testing (EUCAST) (http://www.eucast.org/; [29]).

## Figures and Tables

**Table 1:** Number of screened and the group B streptococcus (GBS) positive samples and the colonization rates measured in the different age groups during the examined period.

Age Groups (Years Old)		<21	21–25	26–30	31–35	36–40	41–45	>45	Total
Year									
2012	No. of screened samples	4	73	468	1037	823	357	114	2876
	No. of GBS positive samples (colonization rate; %)	0 (0)	10 (13.7)	80 (17.1)	196 (18.9)	150 (18.2)	53 (14.8)	17 (14.9)	506 (17.6)
2013	No. of screened samples	6	88	538	966	867	329	116	2910
	No. of GBS positive samples (colonization rate; %)	1 (16.7)	24 (27.3)	96 (17.8)	176 (18.2)	142 (16.4)	52 (15.8)	17 (14.7)	508 (17.5)
2014	No. of screened samples	10	94	539	1015	890	344	115	3007
	No. of GBS positive samples (colonization rate; %)	2 (20.0)	15 (16.0)	106 (19.7)	212 (20.9)	168 (18.9)	69 (20.1)	23 (20.0)	595 (19.8)
2015	No. of screened samples	3	103	557	992	884	352	89	2980
	No. of GBS positive samples (colonization rate; %)	0 (0)	16 (15.5)	101 (18.1)	198 (20.0)	177 (20.0)	67 (19.0)	17 (19.1)	576 (19.3)
2016	No. of screened samples	9	92	568	1001	866	341	89	2966
	No. of GBS positive samples (colonization rate; %)	2 (22.2)	17 (18.5)	123 (21.7)	193 (19.3)	160 (18.5)	57 (16.7)	18 (20.2)	570 (19.2)
2017	No. of screened samples	7	108	584	951	839	331	60	2880
	No. of GBS positive samples (colonization rate; %)	1 (14.3)	14 (13.0)	111 (19.0)	158 (16.6)	147 (17.5)	62 (18.7)	9 (15.0)	502 (17.4)
2018	No. of screened samples	3	75	312	548	480	197	33	1648
	No. of GBS positive samples (colonization rate; %)	0 (0)	13 (17.3)	59 (18.9)	109 (19.9)	85 (17.7)	27 (13.7)	4 (12.0)	297 (18.0)
Total	No. of screened samples	42	633	3566	6510	5649	2251	616	19267
	No. of GBS positive samples (colonization rate; %)	6 (14.3)	109 (17.2)	676 (19.0)	1242 (19.0)	1029 (18.2)	387 (17.2)	105 (17.1)	3554 (18.5)

**Table 2:** Number and proportion of erythromycin and clindamycin resistant strains isolated from the GBS positive samples.

Year	GBS Positive Samples	Erythromycin Resistant Strains (%)	Clindamycin Resistant Strains (%)	Strains Resistant to Erythromycin Alone (%)	Strains Resistant to Clindamycin Alone (%)	Erythromycin-Clindamycin Co-Resistant Strains (%)
2012	506	148 (29.2)	153 (30.2)	6 (1.2)	11 (2.2)	142 (28)
2013	508	172 (33.9)	170 (33.5)	6 (1.2)	4 (0.8)	166 (32.8)
2014	595	190 (31.9)	182 (30.6)	17 (2.9)	9 (1.5)	173 (29.1)
2015	576	222 (38.5)	221 (38.4)	11 (1.9)	10 (1.7)	211 (36.6)
2016	570	196 (34.4)	182 (31.9)	14 (2.5)	0 (0)	182 (31.9)
2017	502	206 (41.0)	206 (41.0)	6 (1.2)	6 (1.2)	200 (39.8)
2018	297	118 (39.7)	115 (38.7)	12 (4.0)	9 (3.0)	106 (35.7)
Total	3554	1239 (34.9)	1230 (34.6)	68 (1.9)	49 (1.4)	1181 (33.2)

**Table 3:** Number and rate of the ST-1 and ST-17 strains detected by the analysis of the matrix-assisted laser desorption/ionization time-of-flight mass spectrometry (MALDI-TOF MS) spectra, and the number and rate of the erythromycin and clindamycin resistant strains among them.

Sequence Type	No. of Strains Detected (%)	Erythromycin Resistant Strains (%)	Clindamycin Resistant Strains (%)	Strains Resistant to Clindamycin Alone (%)	Erythromycin-Clindamycin Co-Resistant Strains (%)
ST-1	71 (27.3)	37 (52.1)	37 (52.1)	0 (0)	37 (52.1)
ST-17	50 (19.2)	16 (32.0)	17 (34.0)	1 (2.0)	16 (32.0)
Other (non-ST-1, non-ST-17)	139 (53.5)	43 (30.9)	47 (33.8)	4 (2.9)	43 (30.9)
